# Hoarders Only Discount Consumables and Are More Patient for Money

**DOI:** 10.3389/fnbeh.2016.00030

**Published:** 2016-03-04

**Authors:** Brian D. Vickers, Stephanie D. Preston, Richard Gonzalez, Andrea M. Angott

**Affiliations:** Department of Psychology, University of Michigan, Ann ArborMI, USA

**Keywords:** hoarding disorder, discounting, impulsivity, addiction, consumption

## Abstract

Individuals with hoarding disorder (HD) excessively acquire and retain goods while also exhibiting characteristics of impulsivity and addiction. However, HD individuals do not always perform impulsively in experiments, they do not appear interested in money, and they exhibit many features of risk-aversion and future-planning. To examine impulsivity in HD, we compared validated community participants high and low in hoarding tendencies on questionnaire measures of hoarding and impulsivity as well as a standard experimental measure of impulsivity (intertemporal discounting) that was modified to compare decisions about money, pens, and snacks. Common discounting effects were replicated. Compared to the low hoarding group, the high hoarding group was more impatient for consumables (pens and snacks) but they were more patient for money. This increased patience for money in high hoarding individuals is in contrast to all other studies on discounting in disordered populations, but consistent with the phenomenology of HD. HD does not appear to be driven by a fundamental inability to wait, but rather a specific, potent desire for consumable rewards.

## Introduction

Individuals with hoarding disorder (HD) are characterized by their excessive acquisition and retention of goods with limited or no value, leading to significantly cluttered living spaces that cannot be used for their original purpose, and significant associated distress and life impairment (Frost and Hartl, 1996). HD now occupies its own diagnostic category in the DSM-V under the OCD and related disorders section (American Psychological Association [APA], 2013), but it is often comorbid with other impulsive–compulsive disorders, including OCD, compulsive buying, gambling, and trichotillomania (Samuels et al., 2002; Frost et al., 2011), leading some to characterize it as one of an extended family of compulsive–impulsive spectrum disorders (e.g., see McElroy et al., 1995). HD also shares many features with addiction, which is also considered by many to be a disorder of impulsivity (reviewed in Bickel and Marsch, 2001; Perry and Carroll, 2008; Odum, 2011). Like addiction, HD involves prioritizing rewarding items over other important life priorities like safety, shelter, and social relationships; it is associated with neural changes in the mesolimbocortical system (reviewed in Wang et al., 2012); and it is difficult to remit even if there are some promising treatment options (Tolin et al., 2015). Thus, despite now occupying its own diagnostic category, transdiagnostic approaches need to continue to understand the role of impulsivity in HD (e.g., Tolin and Villavicencio, 2011; Rasmussen et al., 2013; Timpano et al., 2013).

There is empirical evidence that HD individuals are more impulsive than comparison individuals (comparison groups range across studies from typical community samples to clinical groups with and without hoarding symptoms). However, the evidence is more consistent when individual differences measures are used than when experimental tasks are used. This inconsistency likely reflects the fact that there are multiple, distinct constructs associated with impulsivity including risk seeking, present-focus, response inhibition, loss of control, and delay discounting (see Perry and Carroll, 2008). HD individuals scored more highly on multiple impulsive action and inattention individual difference survey measures (Grisham et al., 2007; Tolin and Villavicencio, 2011; Timpano et al., 2013). They were also more impulsive on a signal detection task despite performing the task more slowly (Grisham et al., 2007) and they had poorer response inhibition on this task, but this appears to be because the HD participants were older than the control participants (Rasmussen et al., 2013). In one study, HD individuals preferred the larger, more immediate reward on the Iowa Gambling Task (Lawrence et al., 2006), but they performed like controls in two other studies using the same task (Grisham et al., 2007; Tolin and Villavicencio, 2011). Thus, evidence for impulsivity in HD is more questionable to date for experimental data, and research has yet to compare HD individuals to controls on a common experimental measure of impulsivity in behavioral economics—the intertemporal discounting task (ITD).

Beyond experimental inconsistencies linking HD to impulsivity, multiple facts question the assumption that HD individuals do have a domain-general problem with impulsivity. HD individuals do not impulsively acquire or keep money and, in fact, they use money especially frugally to obtain goods (Samuels et al., 2008; Frost et al., 2009). The documented paranoid personality traits in HD and their excessive fear of events like home break-ins (Samuels et al., 2008), as well as their indecision and perfectionism (Frost and Gross, 1993), are also characteristics that are more associated with risk-aversion and a future-focus that is unlike that associated with addiction or trait impulsivity (Loewenstein et al., 2001). The goal of hoarding behavior *per se* can also be construed as fundamentally risk-avoidant and future-oriented, as individuals acquire and protect resources that they think they may need later (Frost and Hartl, 1996)—much like the way food-storing animals create, maintain, and protect hoards of food to prepare for future scarcities (Preston and Jacobs, 2001, 2005; Preston, 2013). The hoarding in HD also results in significant interpersonal conflict, discomfort, and difficulty using living spaces (Frost et al., 2000), which could be construed as a short term pain that is being suffered in order to provide a long-term benefit or protection from risk—again unlike impulsivity. Thus, from a global perspective, hoarding behavior actually bears many hallmarks of extreme patience, at least regarding the ultimate goal of the behavior. HD individuals may simply report fears about future needs to mask an irrational impulse to acquire; however, it is also possible that their impulsiveness is a proximate mechanism by which HD individuals achieve a genuine, ultimate goal to provide for the future.

Taken together, impulsiveness in HD needs to be examined empirically, across domains, particularly using the standard, accepted laboratory task for measuring impulsivity in the addiction literature—the ITD. On a typical ITD trial, participants are asked to make a forced choice between accepting a smaller quantity of a reward sooner (e.g., $5 today) vs. a larger quantity of the reward later (e.g., $12 in a month). The exact quantities of the reward sooner vs. later and the precise latency that participants have to wait for each option are systematically altered over many otherwise identical trials so that a “discounting rate” can be calculated per person, over time, representing the degree to which they are susceptible to prefer a smaller amount when delivered sooner—the operationalization of impulsivity.

The current study compared individuals with high and low hoarding tendencies on their intertemporal discounting for goods, food, and money. Most hoarding studies only examine the degree to which individuals acquire or fail to discard material goods; however, we included food because we were interested in the degree to which hoarding reflects an evolved, adaptive food-storing instinct shared with other species (Preston, 2013). We also included money not only because it is the typical unit of reward for ITD tasks in behavioral economics, but also because we hypothesized that high hoarding participants would not be uniformly impulsive, based on anecdotal reports and case studies suggesting that HD individuals are not interested in money *per se*. It is also useful for the large ITD literature to realize that not all individuals are highly motivated by money, which is almost always the only reward provided or compared in behavioral economic studies. Based on our evolutionary view and the phenomenological reports of HD, we predicted that high hoarding individuals would be impulsive for goods and food (pens and snacks), but not money.

## Materials and Methods

### Participants

In order to ensure a broad distribution of hoarding tendencies, participants were recruited using two different advertisements for a decision making study, one that specifically asked for participants who would consider themselves “packrats” and another that did not specify. Of the 38 participants, 27 were community members of any adult age who were compensated $10 for participation (16 high hoarding from the packrat advertisement and 11 low hoarding from the unspecified advertisement). The remaining 11 participants were recruited and compensated with course credit through the university psychology pool (three high hoarding, eight low hoarding). All participants were female, which is common in HD studies (e.g., Frost and Gross, 1993) and practical, given a strong bias for females to respond to community advertisements looking for “packrats” (hereafter referred to as the “high hoarding” group when not discussing the advertisement itself).

This was a study of individual differences rather than the clinical diagnostic category of HD. As such, participants were not given an official clinical interview or diagnosis and they are not referred to here as hoarders or individuals with HD. Instead, we placed participants into high and low hoarding groups based on their response to the advertisements and a validated clinical instrument with high specificity and sensitivity for detecting hoarding with scores >14 on the Hoarding Rating Scale (HRS) (Tolin et al., 2010). The HRS is typically administered in a clinical setting using an interview format, but we have adapted it into an easy-to-administer self-report questionnaire that reliably demonstrates individual differences in the non-clinical population, which also correlate with other individual difference measures that are commonly elevated in HD and with a longer published, validated hoarding questionnaires [i.e., the Savings Inventory-Revised (SI-R); Frost et al., 2004] (see, Wang et al., 2012). To ensure a clear distinction between low and high hoarding participants, rather than just split participants by the advertisement they responded to or whether their HRS scores were above or below of 14, we allowed participants to be placed into the high hoarding group regardless of which advertisement they responded to if they had an HRS scores > 19 (median = 27.00, mean = 26.74, *SD* = 4.96; *n* = 19, mean age = 35.05, *SD* = 17.42) and we allowed participants to be placed into the low hoarding group, regardless of which ad they responded to, if they had an HRS score < 13 (median = 4.00, mean = 4.53, *SD* = 4.03; *n* = 19, mean age = 48.21, *SD* = 15.20). Participants with HRS scores between 13 and 19 were excluded. The two groups did differ statistically in age, *t*(36) = 2.48, *p* = 0.018, $\eta_{p}^{2}$ = 0.146, but in the direction opposite to the predicted confound, as usually HD individuals are older but our high hoarding individuals were younger than our low hoarding individuals. Age was used as a factor in analysis (below) and did not influence the results. High and low hoarding participants did not differ statistically on income, based on results from the Happiness Spending Inventory (Dunn et al., 2008) that we administered for another study completed by the same participants, *t*(36) = 1.04, *p* = 0.306.

Participants were consented and tested individually in the laboratory using MediaLab (Jarvis, 2006) on a Dell desktop PC. All participants were consented and debriefed in writing and in person; all procedures were approved by the Institutional Review Board of the University of Michigan.

### Intertemporal Discounting Task

The ITD task used in this study consisted of three blocks (order randomized) of intertemporal choices about money, pens, or snacks. Units were equilibrated across domains as money was listed in whole dollars and pen and snack choices were worth approximately one dollar each. Participants selected their favorite pen or snack from an array, and all choices thereafter used their chosen item to ensure task interest (e.g., Easy Touch or Precise Gel pens; M&M or Oreo snacks). Each block consisted of 45 choices between a smaller reward (x_1_) sooner (t_1_) or a larger reward (x_2_) later (t_2_) (see **Table 1**). Reward quantities (0–85) and delays (now to 135 days from now) varied across trials.

**Table 1:** Trials in the Intertemporal Discounting Task (ITD), sorted by trial type.

	Options				Analysis Subsection			
				
Trial type	x_1_	t_1_	x_2_	t_2_	Increasing the later reward	Increasing the reward and delay	Exponential discounting	Power utility function
**Small quantities, now or later**								
	1	0	2	16	1			
	1	0	4	16	1			
	1	0	6	16	1			
	1	0	8	16	1			
	1	0	10	16	1			
	0	0	1	7				
	3	0	12	19				
	5	0	8	7				
	10	0	30	40				
**Moderate quantities, now or later**								
	14	0	25	4		1		
	19	0	25	38		2		
	24	0	35	14		3		
	27	0	50	6		4		
	34	0	50	15		5		
	40	0	55	47		6		
	41	0	75	5		7		
	54	0	80	15		8		
	55	0	75	46		9		
**Moderate quantities, now or later (original Kirby items)**								
	14	0	25	19		1		
	19	0	25	53		2	1	
	24	0	35	29		3		
	27	0	50	21		4	3	
	34	0	50	30		5		
	40	0	55	62		6		
	41	0	75	20		7		
	54	0	80	30		8		
	55	0	75	61		9	2	
**Moderate quantities, now or much later**								
	14	0	25	79		1		
	19	0	25	113		2		
	24	0	35	89		3		
	27	0	50	81		4		
	34	0	50	90		5		
	40	0	55	122		6		
	41	0	75	80		7		
	54	0	80	90		8		
	55	0	75	121		9		
**Larger quantities, all delayed**								
	44	32	60	93				1
	55	32	75	93			2	1
	66	32	90	93				1
	32	28	44	90				2
	48	28	66	90				2
	19	20	25	73			1	
	27	15	50	36			3	
	40	28	55	29				
	69	44	85	135				


*Options denoted as in the text with x_*1*_ representing the smaller amount, t_*1*_ the sooner time, x_*2*_ the larger amount, and t_*2*_ the later time; all choices were presented as pairs of (x_1_, t_1_ vs. x_2_, t_2_). Original Kirby items were taken from Kirby et al. (1999). Trials with very small quantities offered now or after a delay were added to their set as were trials that offered their moderate reward amounts with a smaller or a larger delay than they used. New items were added to test specific functional forms and to separate delay from amount. The separate subsections of analysis are presented in the final four columns, with numbers depicting which trials (rows) were relevant for that comparison.*

Before each block participants would select their most preferred stimulus for that domain (e.g., Easy Touch vs. Precise Gel pens in the pen domain). Then, each trial would show them a choice between a smaller amount of their selected item sooner or a larger amount of that same item later, such as 3 Easy Touch pens today or 12 Easy Touch pens in 19 days. The task was self-paced including the length of the break between each of the three blocks. The task within each domain included nine trials used in a prior ITD study of addiction (Kirby et al., 1999), plus 36 more trials that we added to measure responses to smaller and larger quantities and shorter and longer delays than was previously included (described in full below), and to systematically investigate different aspects of the choice attributes. This led to a total of 135 trials for each participant.

Additional trial types were included to expand upon results in four ways. A set of trials at small x_1_, x_2_ values was included to test whether discounting effects are present even for relatively small amounts. Another set of trials systematically varied time and quantity to more precisely estimate how changes in time and quantity influence choice. The final two types of trials investigated whether high or low hoarding participants were more likely to violate assumptions of typical discounting models. Exponential discounting assumes a steadily decreasing likelihood of choosing the larger later (LL) option as the time to acquire it increases at a rate proportional to $\frac{1}{1+t}$, where *t* is time. Power utility is based on the fact that people value increasing amounts less (i.e., diminishing utility); power utility requires that if amounts are both multiplied by a constant, then choices should not change (see **Table 2** for a summary of the specific trials). Because the present study used amounts in three different domains (money, pens, snacks) it is important to distinguish whether patterns of choice are due to changes in the utility of the amount or to changes in the discounting of time. Different discounting patterns could arise when comparing, say, choices for money and choices for pens, not because participants discount time differentially in those two domains but because they value increments in the amounts differentially. The ability to separate utility from discounting comes from our design where we systematically manipulate elements of the amounts and time in each trial.

**Table 2:** Subcategories of trials added to examine different attributes of discounting.

		Variables		
	
Subcategory	Trials	Manipulated	Fixed	Test
Later reward	5	x_2_	x_1_, t_1_, t_2_	Increasing later reward from small x_2_
Delay and moderate reward amounts	27	t_2_	t_1_; x_1_, x_2_	Increasing longer delay
Adding a constant to Time	6	t_1_, t_2_	x_1_, x_2_	Increasing time by the same amount for both ts; Exponential discounting
Multiplying amounts by constant	5	x_1_, x_2_	t_1_, t_2_	Increasing amount by the same amount for both xs; power utility


*Each subcategory is listed below with the number of trials per domain, which variables were manipulated vs. held constant, and the attribute of interest. x_*1*_, smaller amount, x_*2*_, larger amount, t_*1*_, sooner time, t_*2*_, later time.*

### Psychopathological Symptoms

After the ITD, participants completed psychopathology inventories that could be correlated with differences in impulsivity emerging from the task, including the HRS, SI-R, Obsessive Compulsive Inventory-Revised (OCI-R; Foa et al., 2002), Barratt Impulsivity Scale (BIS; Patton and Stanford, 1995), Beck Anxiety Inventory (BAI; Beck et al., 1988a), and Beck Depression Inventory II (BDI; Beck et al., 1988b). All survey measures were administered after the ITD task to avoid priming participants with issues related to their psychopathology before the task.

### Analysis

To assess overall discounting, the more impulsive, SS responses were recoded as 0 and the more patient, LL responses were recoded as 1. Results are presented in terms of the proportion of LL choices, representing the degree of patience or willingness to wait longer for the larger reward. But as is common in the behavioral economics literature, any time a participant or group is referred to as having a higher or steeper discount rate, that means that they value the more patient LL option less in favor of the more impulsive SS option.

All contrasts used the following model except where noted: a logistic curve was fit using a logistic hierarchical linear model (HLM) including fixed factors for group (low hoarding = 0; high hoarding = 1), domain (money, pen, snack), their interaction, and a random factor for each participant’s intercept. Planned contrasts compared discounting money vs. consumables (pens and snacks together) (2, -1, -1) and pens vs. snacks (0, 1, -1). All discounting results remained after separately controlling for age or participant population (community vs. psychology pool), but a few small shifts in result thresholds are noted below under individual difference correlations.

## Results

Analyses are organized by comparing overall discounting rates first, followed by the specific tests for the degree to which choices were influenced by delay and quantity, followed by tests to examine behavioral consistency with both exponential and power law forms of discounting (defined below).

### Overall Discounting

**FIGURE 1:**
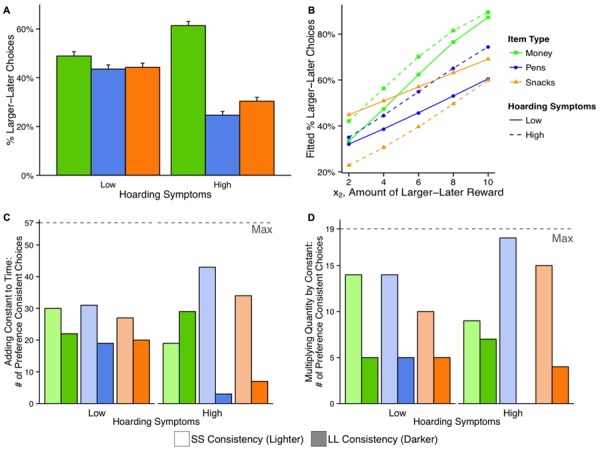
**Intertemporal discounting responses by group (high hoarding vs. low hoarding individuals) and domain (money, pens, snacks).** Percent of participants choosing the larger-later (LL) option overall **(A)**; effects of the increasing later reward amount with a fixed early reward and delay **(B)**; effects of adding a constant to both times **(C)**; effects of a constant multiplier **(D)**. In all plots, choices for money are represented with green coloration, pens in blue, and snacks in orange. For **(B)**, low hoarding individuals are represented by solid lines and high hoarding individuals are represented by dashed lines. For **(C,D)**, the number of times participants made consistent SS choices are in the lighter shade and the number of times participants made consistent LL choices are in the darker shade.

Overall rates of discounting were not different in the two groups, χ^2^(1) = 1.35, *p* = 0.25, but they did differ by domain, χ^2^(2) = 7.97, *p* = 0.021, and the two interacted, χ^2^(2) = 105.76, *p* < 0.001. Money was discounted less than consumables overall, odds ratio (*OR*) = 1.10, *z* = 2.76, *p* = 0.006, and the difference in discounting money compared to pens and snacks was 61% larger in high hoarding than low hoarding participants, money vs. consumables contrast by group: *OR* = 1.61, *z* = 10.17, *p* < 0.001. This interaction was due to high hoarding individuals discounting pens and snacks more steeply than low hoarders and money less steeply than low hoarders, and low hoarders discounting all three domains at similar levels. The contrast between pens and snacks was also marginally larger in high than low hoarders, *OR* = 0.86, *z* = -1.78, *p* = 0.075, with high hoarders responding more impulsively for pens than for snacks, *OR* = 0.85, *z* = -2.81, *p* = 0.005, and low hoarders again treating them similarly, *OR* = 0.98, *z* = -0.35, *p* = 0.729 (see **Figure 1A**). These main effects and interactions were consistent across the subsets of trial types reported below and so are not re-presented in each subsection.

### Effect of the Later Reward Amount with a Fixed Early Reward and Delay

This subset of trials examined the effect on choice of increasing the larger amount (x_2_ = 2, 4, 6, 8, 10) while fixing the smaller amount (x_1_ = 1) and fixing the delay periods (t_1_ = 0, t_2_ = 16 days). The logistic HLM with a centered x_2_ parameter found that people generally waited longer as the amounts increased, x_2_ effect: *OR* = 1.33, *z* = 4.95, *p* < 0.001, and this effect did not differ between high and low hoarding individuals, *OR* = 1.03, *z* = 0.37, *p* = 0.711. As with the overall discounting effects above, as later quantities increased people were still more patient for money than for consumables, domain effect: χ^2^(2) = 7.30, *p* = 0.026; money vs. consumables: *OR* = 1.24, *z* = 2.00, *p* = 0.046, and waited marginally longer for more pens than for more snacks, *OR* = 0.71, *z* = -1.86, *p* = 0.063. At these low reward values, there was no omnibus group difference between money and consumables, group by money vs. consumables: *OR* = 1.22, *z* = 1.29, *p* = 0.196, but high hoarding individuals were more impulsive for snacks than pens while low hoarding individuals were more impulsive for pens than snacks, group by pens vs. snacks: *OR* = 2.13, *z* = 2.97, *p* = 0.003. These slopes across values of x_2_ did not significantly interact by domain and group, x_2_ by domain by group interaction, χ^2^(2) = 0.70, *p* = 0.704. Thus, small and increasing quantities of the larger, later reward (holding the three terms x_1_, t_1_, and t_2_ fixed) reveals no difference in impulsivity for money compared to consumables as x_2_ increases, and at these small amounts high hoarding individuals actually wait longer to acquire more pens than snacks compared to controls (see **Figure 1B**).

### Effect of the Delay and Moderate Reward Amounts with a Fixed Initial Time (Now)

A second subset of trials compared choices between nine original trials (Kirby et al., 1999) to two identical types that varied t_2_ to be earlier or later than the original one. The initial time was fixed to deliver immediate reward (t_1_ = 0), and the early and later reward were moderately sized and fixed within sets of three choices (e.g., x_1_ = 24, x_2_ = 35) (see **Table 1**). HLM analyses compared the effect of the delay within sets of three trials (original t_2_, earlier t_2_, later t_2_) and the effect of the total amount of reward across the nine sets of three trials. A parameter was added to the HLM for t_2_, ordering each of the nine triplets by their mean x_1_, x_2_ amounts to assess quantity effects with a centered linear “quantity contrast.” This analysis complements the previous one by changing which variable was manipulated while leaving the others constant. Previously we only varied the second quantity and this time we only varied the length of the larger delay interval while holding the other three parameters fixed.

**FIGURE 2:**
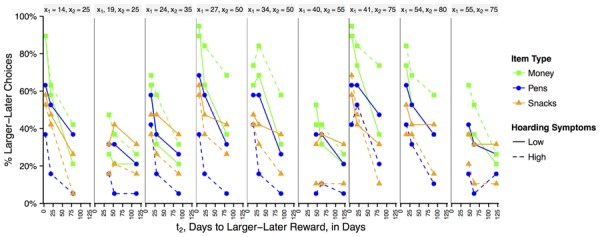
**Intertemporal discounting responses for effects of delay and moderate reward amounts with a fixed initial time (now).** The percent of participants choosing the LL option overall split by smaller-sooner (x_1_) and larger-later (x_2_) reward amounts, which are listed above plots. Choices for money are represented with green lines, pens in blue, and snacks in orange. Low hoarding individuals are represented by solid lines and high hoarding individuals are represented by dashed lines.

As the t_2_ delay increased, participants generally became increasingly impatient for money compared to consumables, t_2_ by money vs. consumables: *OR* = 0.99, *z* = -4.47, *p* < 0.001, and more impatient for pens than snacks, t_2_ by domain omnibus: χ^2^(2) = 26.85, *p* < 0.001; t_2_ by pens vs. snacks: *OR* = 0.994, *z* = -2.54, *p* = 0.011. But looking separately at each group, only low hoarding individuals showed this increasing impatience with longer delays for money and pens, t_2_ by group by domain: χ^2^(2) = 11.77, *p* = 0.003; t_2_ by domain in high hoarding individuals: χ^2^(2) = 0.37, *p* = 0.831; t_2_ by domain in low hoarding individuals: χ^2^(2) = 27.03, *p* < 0.001; t_2_ by money vs. consumables in low hoarding individuals: *OR* = 0.993, *z* = -4.48, *p* < 0.001; t_2_ by pen vs. snack in low hoarding individuals: *OR* = 0.994, *z* = -2.55, *p* = 0.011. There were no effects of the amount of moderate reward or any other effects in this model, χ^2^s < 2.55, *p*s > 0.115 (see **Figure 2**). Therefore, low hoarding individuals had high discount rates for money compared to consumables (i.e., would only choose LL after short delays for money) but high hoarding individuals had similar discount rates for both money and consumables as t_2_ increases.

### Effects of Adding a Constant to Both Times: Testing Exponential Discounting

Theories of exponential discounting hold that people’s discounting rates decline exponentially as time increases, and the rate of discounting only depends on the difference in time between the two points. Exponential discounting assumes that with fixed quantities, delaying both t_1_ and t_2_ by the same constant will not alter choices. To assess the fit of exponential curves, crosstabs were created in the form (x_1_, t_1_ vs. x_2_, t_2_) compared to (x_1_, t_1_ + *k* vs. x_2_, t_2_ + *k*), where *k* is a constant.

The number of preference reversals were summed over the three trial pairs (i.e., 0, 1, 2, or 3 preference reversals) and compared with a Poisson HLM, but this time using the number of preference reversals as the dependent variable. The original trials involved moderate reward amounts (19–75 items) offered immediately (0 days) or after a moderate to large delay (21–93 days), and to these trials a corresponding constant of 15, 20, or 32 days was added to both the early and the later option.

Overall, choices were consistent across groups and domains, χ^2^(1)s < 0.348, *p*s > 0.50. Within each domain, there was also no difference in the number of consistent compared to inconsistent choices between the low and high hoarding groups, *z*s < 0.20, *p*s > 0.60. In line with the high hoarding group’s general consummatory impulsiveness, high hoarding individuals predominantly made consistently impulsive choices for pens and snacks (about eight times more often SS–SS than LL–LL) but chose more often to be consistently patient for money (50% more LL–LL than SS–SS). The low hoarding individuals, instead, were biased to be somewhat impulsive for all three domains, choosing SS–SS about 50% more often than LL–LL for pens, snacks, and money (see **Table 3**; **Figure 1C**). Thus, both low and high hoarding groups were consistent with assumptions of exponential discounting, but high hoarding individuals had many more SS–SS consistent choices for pens and snacks compared to low hoarding individuals, as was the case in the overall discounting rates.

**Table 3:** Cross-tabulations by group and domain for tests of exponential discounting, with a constant added to both times with (x_1_, t_1_ vs. x_2_, t_2_) compared to (x_1_, t_1_ + k vs. x_2_, t_2_+k).

**Low hoarding individuals, money**				**High hoarding individuals, money**			
	
		(x_1_, t_1_ + k) vs. (x_2_, t_2_ + k)				(x_1_, t_1_ + k) vs. (x_2_, t_2_ + k)	
(x_1_, t_1_) vs. (x_2_, t_2_)		SS	LL	(x_1_, t_1_) vs. (x_2_, t_2_)		SS	LL
	SS	30	4		SS	19	5
	LL	1	22		LL	4	29
	
**Low hoarding individuals, pens**				**High hoarding individuals, pens**			
	
		(x_1_, t_1_+ k) vs. (x_2_, t_2_+ k)				(x_1_, t_1_+ k) vs. (x_2_, t_2_+ k)	
(x_1_, t_1_) vs. (x_2_, t_2_)		SS	LL	(x_1_, t_1_) vs. (x_2_, t_2_)		SS	LL
	SS	31	3		SS	43	9
	LL	4	19		LL	2	3
	
**Low hoarding individuals, snacks**				**High hoarding individuals, snacks**			
	
		(x_1_, t_1_ + k) vs. (x_2_, t_2_ + k)				(x_1_, t_1_ + k) vs. (x_2_, t_2_ + k)	
(x_1_, t_1_) vs. (x_2_, t_2_)		SS	LL	(x_1_, t_1_) vs. (x_2_, t_2_)		SS	LL
	SS	27	7		SS	34	10
	LL	3	20		LL	6	7


*Each 2 × 2 Table is based on three trials per participant (each 2 × 2 table has 57 entries).*

### Effects of Multiplying a Constant by Both Amounts: Testing the Power Utility Function

The power utility function can produce effects that may be similar to discounting. If people have different utilities for, say, money and pens, then we may incorrectly attribute the difference to discounting rather than utility. Power utility assumes that when both times in the same trial are multiplied by a constant, *k*, the preferences will not change.

We compared trials that were identical except for a constant *k* multiplier that was applied to both smaller (x_1_) and larger (x_2_) reward amounts; e.g., (x_1_, t_1_ vs. x_2_, t_2_) compared to (*k* ● x_1_, t_1_ vs. *k* ● x_2_, t_2_). These trials all involved larger quantities of items in both smaller and larger positions (i.e., from 32 to 90) offered at two delays that were both displaced in time and never immediate (32 vs. 93 days or 29 vs. 90 days). This set of trials included one pair and one triplet. The trial pair used *k* = 1.38 with x_1_, x_2_ pairs of (32, 44) and (48, 66). The triplet used *k* = 1.36 with x_1_, x_2_ pairs of (44, 60), (55, 75), and (66, 90). Again, the Poisson test was used to compare the number of preference reversals for each domain (0, 1, 2, or 3 of the possible 3 reversals from SS to LL or from LL to SS). Supporting the power utility function, choices on these trials were consistent in both low and high hoarding groups, across domains, χ^2^(1) < 1.96, *p*s > 0.373. Within each domain, there was also no difference in the number of preference reversals between low and high hoarding groups, *z*s < |1.20|, *p*s > 0.250. The nature of the consistent choices in this set of trials was similar to that of the prior section, with high hoarding individuals being overwhelmingly, consistently impatient for pens and snacks (SS–SS, with actually no LL–LL choices for high hoarding individuals deciding about pens), but more often consistently patient for money. Conversely, the low hoarding group was biased to be consistent in all three domains by being two to three times more impatient than patient (see **Table 4**; **Figure 1D**).

**Table 4:** Cross-tabulations by group and domain for tests of the power utility function, with a constant added to both amounts with (x_1_, t_1_ vs. x_2_, t_2_) compared to (k ● x_1_, t_1_ vs. k ● x_2_, t_2_).

**Low hoarding individuals, money**				**High hoarding individuals, money**			
	
		(k ● x_1_,t_1_) vs. (k ● x_2_,t_2_)				(k ● x_1_,t_1_) vs. (k ● x_2_,t_2_)	
(x_1_, t_1_) vs. (x_2_, t_2_)		SS	LL	(x_1_, t_1_) vs. (x_2_, t_2_)		SS	LL
	SS	14	0		SS	9	2
	LL	0	5		LL	1	7
	
**Low hoarding individuals, pens**				**High hoarding individuals, pens**			
	
		(k ● x_1_,t_1_) vs. (k ● x_2_,t_2_)				(k ● x_1_,t_1_) vs. (k ● x_2_,t_2_)	
(x_1_, t_1_) vs. (x_2_, t_2_)		SS	LL	(x_1_, t_1_) vs. (x_2_, t_2_)		SS	LL
	SS	14	0		SS	18	0
	LL	0	5		LL	1	0
	
**Low hoarding individuals, snacks**				**High hoarding individuals, snacks**			
	
		(k ● x_1_,t_1_) vs. (k ● x_2_,t_2_)				(k ● x_1_,t_1_) vs. (k ● x_2_,t_2_)	
(x_1_, t_1_) vs. (x_2_, t_2_)		SS	LL	(x_1_, t_1_) vs. (x_2_, t_2_)		SS	LL
	SS	10	4		SS	15	0
	LL	0	5		LL	0	4


*Consistent preferences are demonstrated by higher values in SS/SS and LL/LL cells (diagonals in quadrants one and three), indicating that participants decided similarly in the original decision and the *k* shifted version. This trial type included one pair and one triplet but to avoid overlapping choices from the triplet only the pair is presented here.*

### Individual Differences Measures

As a complementary analysis to the group-level analysis above, we also investigated whether continuous, individual-level variation in the degree to which participants exhibited hoarding, impulsivity, and psychopathological tendencies correlated with their main outcome variable from the discounting analysis reported above. For each person we correlated their individual difference data across all of the scales with the degree to which they were more patient for money than consumables by subtracting their pooled LL choices for both pens and snacks from their percent of LL choices for money. This produced a variable ranging from +1 (100% choices for money and 0% choices for pens and snacks) to -1 (0% choices for money and 100% LL choices for pens and snacks), with the zero point representing similar proportions for money and consumables. Consistent with the group-level effects above—where high hoarding individuals were more patient for money than consumables—this behavior was also significantly correlated with the continuous, individual difference measures across the whole sample including all hoarding scales (HRS, SI-R Clutter, SI-R Difficulty Discarding, SI-R Acquisition, OCI-R Hoarding), *r*s(36) > 0.33, *p*s ≤ 0.05, $\eta_{p}^{2}$s > 0.10, and the BIS Attentional Impulsivity subscale, *r*(36) = 0.40, *p* = 0.012, $\eta_{p}^{2}$ = 0.16 (see **Table 5**). Even after controlling for age and population (community vs. or psychology pool) all results remained except that scores from the pre-screening administration of the HRS became marginal in both cases and the correlations with SI-R acquisition dropped to the marginal level, *t*s(35) > 1.77, *p*s < 0.09, $\eta_{p}^{2}$ s > 0.08.

**Table 5:** Correlations of psychopathology measures with the main effect of the intertemporal discounting task (ITD).

	% LL Difference	
	Money – Snack and Pen	
Scale	*r*	*p*
**Hoarding scales**		
HRS pre-screen	0.33	0.04^∗^
HRS post	0.37	0.02^∗^
SIR clutter	0.35	0.03^∗^
SIR difficulty discarding	0.39	0.02^∗^
SIR acquire	0.36	0.03^∗^
OCI-R hoarding	0.36	0.03^∗^
**Non-hoarding psychopathology scales**		
BAI	0.13	0.44
BDI	0.27	0.10^t^
STAIT	0.13	0.44
**Impulsivity subscales**		
BIS attention	0.40	0.01^∗^
BIS motor	0.15	0.38
BIS non-planning	0.16	0.35


*High hoarding individuals discounted money less and consumables (pens and snacks) more than low hoarding individuals, so individual differences measures were correlated with the difference score of the % larger-later (LL) decisions for money minus pens and snacks. All correlations have *df* = 36. HRS, Hoarding Rating Scale. SIR, Savings Inventory Revised. OCI-R, Obsessive Compulsive Inventory, Revised. BAI, Beck Anxiety Inventory. BDI, Beck Depression Inventory. BIS, Behavioral Impulsivity Scale. STAIT, State Trait Anxiety Inventory, State. ^∗^*p* < 0.05, ^*t*^*p* < 0.10.*

## Discussion

Using a modified version of a standard impulsivity task from behavioral economics, we found reliable evidence across multiple measures and analysis strategies that people with problematic hoarding tendencies are actually more patient than people with low hoarding tendencies for money, but they are indeed less patient for consumable goods, particularly pens. The high hoarding group’s greater patience for money suggests that they do not have a domain-general problem with impulsivity as has been suggested for addiction (e.g., Odum, 2011), and that they do possess the cognitive capacity to save or wait for larger reward.

To our knowledge, this is the first time a disordered population has demonstrated greater patience for money compared to controls, or to have shown discounting rates for money that are inversely correlated with discounting for consumable rewards. Extensive prior research finds that impaired populations—including those addicted to heroin, cocaine, alcohol, cigarettes, food and gambling—discount their drug of choice more steeply than money while also discounting money more steeply than controls, even compared to ex-users (see reviews in Bickel and Marsch, 2001; Perry and Carroll, 2008). The fact that drugs are usually discounted more steeply than money is attributed to the more direct impact of consumables on the biological reward system, with many studies finding greater discounting for consumables like food, candy, or beer compared to money, even in non-disordered populations (Odum and Rainaud, 2003; Odum et al., 2006; Estle et al., 2007). We replicated this overall effect of greater discounting for consumables, but also found that participants with low tendencies to hoard treated the three domains more similarly to one another. However, the group with high hoarding tendencies discounted money less steeply and discounted consumables more steeply than controls. Thus, domain-specific discounting rates may be more powerful in populations with a focused desire. This differential treatment of money by individuals with high hoarding tendencies does accord with their real-world apparent lack of interest in making and accumulating money and their frugal use of it to obtain desired goods (Frost et al., 2009).

Our results also suggest that the delay impacts choice more than the quantity, particularly for rewards that are offered “now.” The participants with high hoarding tendencies were particularly prone to impulsively obtain pens—even more so than snacks—again attesting to their domain-specific interest in goods *per se*. This is particularly interesting in light of prior studies across domains that presumed that drugs of abuse and food are discounted more steeply than money because they can be consumed (and, thus, activate the biological reward system more strongly). For the participants with high hoarding tendencies, pens are their desired item of choice and they appear to strongly drive the choice system despite not being literal consummatory reward. However, paradoxically, the individuals with high hoarding tendencies did prefer a single snack delivered immediately over more snacks delivered later while being willing to wait for a delay from now to accumulate more than one pen; they also generally preferred multiple pens immediately over more pens later. Such reversals perhaps make sense if you either consider that only single snacks can be consumed immediately (and thus have a greater appeal to people with hoarding tendencies only in cases when t_1_ = 0) or if wanting to accumulate material goods takes a non-linear function that eventually curves downward, whereby more pens is better but there are limits to how many you could need or how many in the short term could satisfy the desire. Regardless, these complexities attest to the need for future ITD work to include a broad range of relevant units and delays to capture important biologically-relevant stages of the process and units of interest (see also Odum et al., 2006).

The particular impatience of high hoarding individuals for immediate consumables suggests that the “incentive salience” (cf., Berridge and Robinson, 2003) of goods is what makes the items difficult for these people to resist (see an overview of the biological mechanisms for cross-domain reward in Preston et al., 2014). Similarly, in a prior study, neural activity in the nucleus accumbens—the region associated with the motivation to acquire rewarding drugs—increased during the acquisition of goods to the extent that participants reported real-world problems with hoarding (Wang et al., 2012). This rewarding property of goods for people with HD, but not for money, has been underappreciated—with most theories focusing on their indecision or fear of making mistakes (e.g., see Frost and Hartl, 1996). Perhaps fear can potentiate a proximate motivation toward goods to alleviate future uncertainty, to provide comfort in the absence of rewarding social bonds, or to provide literal, physical protection (Preston, 2013).

Multiple facts suggest that our task and results are valid. Unlike in some other experimental tests of HD and impulsivity, we did not find null effects and we replicated most standard discounting effects, including greater patience for increasing later reward and greater impatience for immediate, early reward and for consumables over money. We also replicated our results from the two group comparison of high vs. low hoarding individuals with continuously-varying individual difference correlations across the whole sample; these correlations also replicate a prior study that found greater attentional impulsivity in HD (Tolin and Villavicencio, 2011). Our results also remained similar after controlling for age and recruitment method (community or psychology pool). Moreover, our results affirmed the assumptions of exponential discounting and the power utility function, particularly in low hoarding participants. The individuals with high hoarding tendencies did tend to be consistent for consumables when a constant was added to both delays (which does not violate exponential discounting), but only because they preferred smaller amounts of pens or snacks offered immediately while preferring the larger quantity when the two time periods were shifted away from “now,” which is consistent with the known potency of immediately available reward.

In addition to replicating many common findings in the delay discounting literature, our task and trial design used an enhanced version of a standard set of delay discounting items (Kirby et al., 1999) with items that allowed us to test for specific forms of discounting. For instance, exponential discounting requires that if a person chooses (x_1_, t_1_) over (x_2_, t_2_) then they should choose (x_1_, t_1_ + *k*) over (x_2_, t_2_ + *k*). Evidence of preference reversals across such a pair rejects exponential discounting without requiring that we estimate parameter values (see Krantz et al., 1971, for an explanation of this approach). This approach allows us to determine whether exponential discounting holds across money, pens, and snacks, and whether it holds equally in the two groups. We opted not to perform non-linear parameter estimation because it would be difficult to compare across three domains. For example, if we fit a hyperbolic discounting function and observed differences across the three domains in the discounting parameter, we would not be able to determine whether the different parameters actually resulted from differences in discounting across the domains or from a confounding difference in utility (e.g., because a pack of cookies really isn’t worth an exact dollar or means something different when you have 5 packs or 35 packs compared to 1). The approach adopted in this study tailored the trial types to study different aspects of the decision while making fewer assumptions about the domains and using statistically powered but simple tests that allow us to compare groups and domains. A more careful simultaneous assessment of discounting vs. utility functions across domains is beyond the scope of this project, but should also be investigated.

Despite the novelty and consistency of our results, there are some limitations. Our choices were not incentivized with real rewards and our high hoarding group was identified through self-report, which was followed up with a validated clinical questionnaire rather than a structured clinical interview. However, ITD procedures are known to produce similar results whether the rewards are hypothetical or real (Johnson and Bickel, 2002; Madden et al., 2003) and hoarding should be thought of as a continuously varying individual difference and not just a present or absent psychopathology (reviewed in Preston et al., 2009; Preston, 2013). One must also not assume that greater attentional impulsivity and ITD discounting for goods should be extended to other forms of impulsivity that we did not measure, or other aspects of HD such as discarding problems. In clinical samples up to 60% of hoarding patients meet the criteria for compulsive buying (e.g., Frost et al., 2002), so it is possible that this impulsiveness for consumable reward explains the compulsive buying and acquisition of free items in HD, but we need to determine empirically which if any other forms of impulsiveness are disordered in HD. A large, systematic study that compares all forms of impulsivity within participants and across groups and domains is still needed.

A trans-diagnostic, symptom-based approach to HD that generously includes both empirically validated tasks and individual difference measures can help us understand the underlying problems that promote HD. This approach can also help us understand the potential link between HD and other related phenomena like impulse-control disorders and addiction, which is currently poorly understood and likely prevents us from being maximally effective in treating HD.

## Author Contributions

SP developed the study concept. SP, RG, and AA designed the study. SP and AA oversaw subject recruitment and testing. BV performed data analysis under the supervision of SP and RG. SP and BV drafted the paper, and all authors provided revisions and approved the final version of the paper for submission.

## Conflict of Interest Statement

The authors declare that the research was conducted in the absence of any commercial or financial relationships that could be construed as a potential conflict of interest.
